# The inflammatory phase of fracture healing is influenced by oestrogen status in mice

**DOI:** 10.1186/s40001-017-0264-y

**Published:** 2017-07-06

**Authors:** Melanie Haffner-Luntzer, Verena Fischer, Katja Prystaz, Astrid Liedert, Anita Ignatius

**Affiliations:** Institute of Orthopaedic Research and Biomechanics, University Medical Centre Ulm, Helmholtzstraße 9, 89081 Ulm, Germany

**Keywords:** Fracture healing, Oestrogen, Inflammation, Midkine, Neutrophils

## Abstract

**Background:**

Fracture healing is known to be delayed in postmenopausal, osteoporotic females under oestrogen-deficient conditions. Confirming this, experimental studies demonstrated impaired callus formation in ovariectomised animals. Oestrogen-deficiency is known to affect the immune system and the inflammatory response during wound healing. Because a balanced immune response is required for proper bone healing, we were interested to ascertain whether the early immune response after facture is affected by oestrogen depletion.

**Methods:**

To address the above question, female mice received either a bilateral ovariectomy (OVX) or were sham-operated, and femur osteotomy was performed 8 weeks after OVX/sham operation. The effects of OVX on the presence of immune cells and pro-inflammatory cytokines were evaluated by flow cytometry and immunohistochemistry of the fracture calli on days 1 and 3 after fracture.

**Results:**

One day after fracture, immune cell numbers and populations in the fracture haematoma did not differ between OVX- and sham-mice. However, on day 3 after fracture, OVX-mice displayed significantly greater numbers of neutrophils. Local expression of the oestrogen-responsive and pro-inflammatory cytokine midkine (Mdk) and interleukin-6 (IL-6) expression in the fracture callus were increased in OVX-mice on day 3 after fracture compared with sham-mice, indicating that both factors might be involved in the increased presence of neutrophils. Confirming this, Mdk-antibody treatment decreased the number of neutrophils in the fracture callus and reduced local IL-6 expression in OVX-mice.

**Conclusions:**

These data indicate that oestrogen-deficiency influences the early inflammatory phase after fracture. This may contribute to delayed fracture healing after oestrogen depletion.

## Background

There is clinical evidence for a prolonged fracture healing time in postmenopausal, osteoporotic females [1, 2]; however, the pathomechanisms are currently not fully understood. Confirming clinical data, experimental studies demonstrated that ovariectomised rats and mice display impaired cartilaginous callus formation and reduced vascularisation during fracture healing [3–5]. In the late phase of healing, oestrogen-deficiency decreased the amount of the newly formed bone and the mechanical competence of the fracture callus [6–9]. On a cellular level, both osteoblast and osteoclast numbers were significantly increased, indicating high bone turnover with a shift towards bone resorption [10]. These studies indicate that osteoporotic bone healing is delayed due to impaired angiogenesis and cartilage formation, and an imbalance of osteoblast and osteoclast activities.

Oestrogen-deficiency also affects the immune system. Postmenopausal females display a pro-inflammatory phenotype with increased numbers of activated T-cells and B-lymphocytes [11] and higher levels of circulating pro-inflammatory cytokines, including interleukin-1 (IL-1), IL-6, IL-31 and tumour necrosis factor α (TNFα) [12–14]. Furthermore, the immune response is altered when the system is challenged. For example, the inflammatory response was increased in oestrogen-deficient mice after induction of paw inflammation or rheumatoid arthritis [15–17]. During wound healing, pro-inflammatory cytokines were up-regulated in rodents subjected to ovariectomy (OVX), resulting in delayed skin repair [18, 19].

A balanced immune response is regarded to be crucial also for successful bone healing, because it was shown that bone regeneration is disturbed under local and systemic inflammatory conditions [20–22]. However, the influence of oestrogen-deficiency on the inflammatory response in fracture healing has not yet been investigated, despite the high clinical relevance of delayed bone regeneration in postmenopausal osteoporosis. Our own previous work provided evidence that oestrogen-deficiency may affect the inflammatory response to fracture. We found that after bone fracture, OVX-mice displayed increased serum levels of midkine (Mdk) [23], a pro-inflammatory cytokine and a negative regulator of bone remodelling [24–26]. *Mdk* is an oestrogen-responsive gene and its expression is known to be up-regulated in the absence of oestrogen [27, 28] as well as during inflammatory diseases and tissue injury and regeneration [29–33]. The absence of Mdk reduced leukocyte recruitment to the sites of inflammation during nephritis, arthritis and other inflammatory diseases [33].

Therefore, we hypothesised that oestrogen-deficiency alters the early inflammatory response after fracture and that inflammatory mediators, including Mdk, may be involved in this effect. To test these hypotheses, we analysed the presence of immune cells and inflammatory cytokines in the fracture haematoma of OVX-mice as well as the impact of treatment with an antibody targeting Mdk.

## Methods

### Animal experiments

All animal experiments were in compliance with international regulations for the care and use of laboratory animals with the approval of the local ethical committee (No. 1079 and 1184, Regierungspräsidium Tübingen, Germany). Female C57BL/6J mice were maintained in groups of two to four animals per cage (370 cm^2^) on a 14-h light and 10-h dark circadian rhythm with water and food ad libitum. Mice aged 3–4 months underwent bilateral sham operation or OVX as described previously [34]. Osteotomy was performed 8 weeks after sham/OVX. The surgery was conducted according to the published protocol [35]. Briefly, the M. biceps femoralis and the M. vastus lateralis at the right femur were separated bluntly to minimise additional soft tissue trauma. A semi-rigid external fixator (axial stiffness of 3 N/mm) was mounted parallel to the femur shaft with four screws. The osteotomy was created in the middle of the femur diaphysis using a 0.4-mm gigli wire saw, and muscles and skin were adapted.

All mice were fed a phytoestrogen-free diet for the entire period of the experiments. Mice were euthanised on day 1, 3 or 23 after fracture using carbon dioxide. The uteri and fractured and intact femurs were removed for further analysis.

For evaluation of the effects of Mdk on the fracture callus, sham- and OVX-mice received a subcutaneous injection with 25 mg/kg Mdk-antibody (Mdk-Ab) directly after the osteotomy surgery, as described previously [23]. The mouse-anti human Mdk IgG1 monoclonal antibody was shown to be cross-reactive to murine Mdk (ELISA EC_50_ to human Mdk: 31.7 ng/ml and ELISA EC_50_ to murine Mdk: 37.8 ng/ml) [36] and to block Mdk in the serum of fractured mice with an efficiency between 65 and 100% depending on the measurement time point [23, 36]. Mice were euthanised on day 3, and fractured femurs were subjected to decalcified histology.

### Oestrogen ELISA

Sera of sham- and OVX-mice euthanised 1 day after fracture were tested for oestrogen concentrations using the commercially available oestrogen ELISA according to the manufacturer’s instructions (Calbiotech #ES180S-100, Spring Valley CA, USA).

### Micro-computed tomography (µCT)

Femurs of mice sacrificed on day 23 were analysed using a µCT scanning device (Skyscan 1172, Bruker, Kontich, Belgium) operating at a voxel resolution of 8 µm (50 kV, 200 mA). Bone mineral density (BMD) was assessed using two phantoms each with a defined density of hydroxyapatite (250 and 750 mg/cm^3^) within each scan. Discrimination between non-mineralised and mineralised tissue was performed using a global threshold of 642 mg hydroxyapatite/cm^3^ according to Morgan et al. [37] and in accordance with the American Society for Bone and Mineral Research (ASBMR) guidelines for µCT analysis [38]. Three-dimensional (3D) reconstruction of the fracture callus between the two inner pin holes was performed using CTvol software (Bruker).

### Fluorescent-activated cell sorting (FACS) analysis

To analyse inflammatory cells in the fracture haematoma, FACS analysis was performed. On day 1 after fracture, the fractured femur and the contralateral bone marrow were harvested. The fracture haematoma was collected using a surgical scissor. The contralateral bone marrow was flushed out using phosphate-buffered saline. The fracture haematoma was passed through a 70-µm cell strainer (Corning Inc., Durham, NC, USA) to obtain a single-cell suspension and the cells of the fracture haematoma and bone marrow were subjected to erythrolysis. For the identification of macrophages (Ly6G^−^, F4/80^+^, CD11b^+^), neutrophils (Ly-6G^+^, F4/80^−^, CD11b^+^), inflammatory monocytes (F4/80^+^, Ly-6G^+^, CD11b^+^), B-lymphocytes (CD19^+^), T-lymphocytes (CD3^+^), cytotoxic T-lymphocytes (CD3^+^, CD8^+^), and T-helper-lymphocytes (CD3^+^, CD4^+^), the antibodies listed in Table 1 were used. Specific isotype-matched immunoglobulin antibodies (Table 1) were used as negative controls. Cells (fracture haematoma: totality of cells isolated; bone marrow: 1 × 10^6^ cells) were incubated with the antibodies for 30 min on ice. 7-aminoactinomycin (7-AAD, Sigma, Steinheim, Germany) was used for dead-cell discrimination. Cells were analysed using an LSR II flow cytometer (BD Bioscience) and FlowJo software v10 (FlowJo LLC, Ashland, OR).

**Table 1:** Antibodies used for flow cytometry

Antibody	Label	Product	Company	Dilution
Il-6 (rabbit anti-mouse)	–	bs-0782R	Bioss	1:250
Mdk (goat anti-mouse)	–	sc-1398	SantaCruz	1:100
CCL2 (rabbit anti-mouse)	–	bs-1955R	Bioss	1:150
CXCL1 (rabbit anti-mouse)	–	ab86436	Abcam	1:200
Ly6G (rat anti-mouse)	–	127603	BioLegend	1:300
CD45/B220 (rat anti-mouse)	–	RA3-6B2	BioLegend	1:100
CD8 (rabbit anti-mouse)	–	bs-0648R	Bioss	1:100
F4/80 (rat anti-mouse)	–	ab6640	AbD Serotec	1:500
Streptavidin	HRP	ZUC012	Zytomed Sytems	1:100
IgG (donkey anti-goat)	Biotin	sc-3854	SantaCruz	1:100
IgG (goat anti-rabbit)	Biotin	B2770	Life Technologies	1:100
IgG (goat anti-rat)	Biotin	31830	Invitrogen	1:100

### Immunohistochemistry and immunofluorescence staining

Femurs of mice sacrificed 3 days post-surgery were fixed in 4% formalin, decalcified using 20% ethylenediaminetetraacetic acid (pH 7.2–7.4) for 10–12 days and embedded in paraffin after dehydration in an ascending ethanol series. Longitudinal cross-sections with a thickness of 7 μm were prepared. Immunohistochemical and immunofluorescence staining of IL-6, Mdk, CCL2, CXCL1, Ly6G (neutrophils), CD45R (B-lymphocytes), CD8 (cytotoxic T-lymphocytes) and F4/80 (macrophages) were performed using the antibodies specified in Table 2. Species-specific non-targeting immunoglobulins were used as isotype controls. 3-Amino-9-ethylcarbazol (Zytomed Systems) was used as the chromogen and the sections were counterstained using haematoxylin (Waldeck, Münster, Germany). FITC-streptavidin was used for immunofluorescence staining and the sections were counterstained using DAPI. The sections were examined by light or fluorescence microscopy (DMI6000 B, Leica, Heerbrugg, Switzerland). The amount of callus tissue and the number of positively stained cells were determined by image analysis software (Leica MMAF 1.4.0 Imaging System, Leica).

**Table 2:** Antibodies used for immunohistochemistry

Antibody	Label	Product	Company	Dilution
CD3e (Armenian hamster anti-mouse)	PE-Cyanine 7	25-0031	Affymetrics eBioscience	1:100
CD4 (rat anti-mouse)	APC-eFlour^®^ 780	47-0041	Affymetrics eBioscience	1:200
CD8a (rat anti-mouse)	APC	17-0081	Affymetrics eBioscience	1:800
CD11b (rat anti-mouse)	Alexa Flour^®^ 700	56-0112	Affymetrics eBioscience	1:400
CD19b (rat anti-mouse)	PE	12-0193	Affymetrics eBioscience	1:400
F4/80 (rat anti-mouse)	FITC	11-4701	Affymetrics eBioscience	1:50
Ly-6G (rat anti-mouse)	V450	560603	BD Bioscience	1:400
IgG Isotype (Armenian hamster)	PE-Cyanine 7	25-4888	Affymetrics eBioscience	1:100
IgG2b K Isotype (rat)	APC-eFlour^®^ 780	47-4031	Affymetrics eBioscience	1:200
IgG2a K Isotype (rat)	APC	17-4321	Affymetrics eBioscience	1:800
IgG2b K Isotype (rat)	Alexa Flour^®^ 700	56-4031	Affymetrics eBioscience	1:400
IgG2a K Isotype (rat)	PE	12-4321	Affymetrics eBioscience	1:400
IgG2a K Isotype (rat)	FITC	11-4321	Affymetrics eBioscience	1:50
IgG2a K Isotype (rat)	V450	560377	BD Bioscience	1:400

### Statistics

Statistical analysis was performed using Shapiro–Wilk test for normal distribution and ANOVA/LSD post hoc test with SPSS software (SPSS Inc., Chicago, IL, USA). All results are presented as the mean and standard deviation. Values of *p* < 0.05 were considered as statistically significant. Sample size was *n* = 5–6 per group and each time point.

## Results

### OVX-induced osteoporosis and delayed fracture healing

**Fig. 1:**
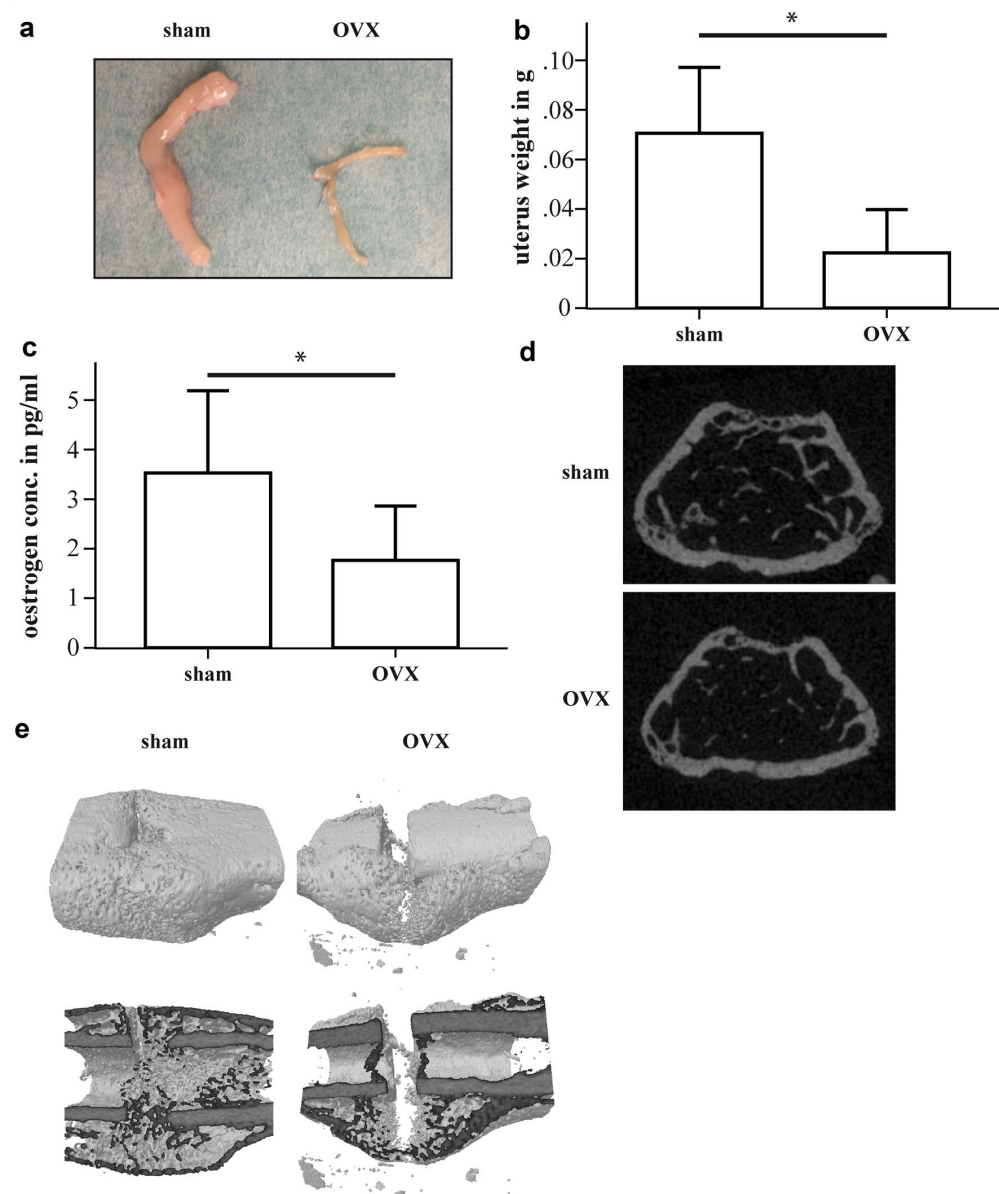
Influences of ovariectomy (OVX) on uterus weight, intact bone and bony callus formation. **a** Uteri from sham-operated and OVX-mice 11 weeks after initial surgery. **b** Weights of the uteri. **c** Oestrogen serum levels at day 1 after osteotomy surgery. **d** Representative transversal µCT images of the metaphyseal region of the intact femurs. **e** Representative longitudinal 3D µCT images of the fracture calli of sham-operated and OVX-mice on day 23 after fracture

We first confirmed the known effects of OVX on the skeleton and on fracture healing [3, 39]. As expected, uteri of mice subjected to castration displayed severe atrophy (Fig. 1a) and a significantly decreased weight (Fig. 1b). Further, oestrogen serum levels were significantly reduced in OVX-mice (Fig. 1c). µCT analysis of the intact femurs demonstrated an osteoporotic phenotype in the trabecular compartment (Fig. 1d). Analysis of the fracture calli on day 23 showed considerably disturbed bony callus development and poor cortical bridging due to OVX (Fig. 1e).

### OVX increased the number of neutrophils in the fracture haematoma

**Fig. 2:**
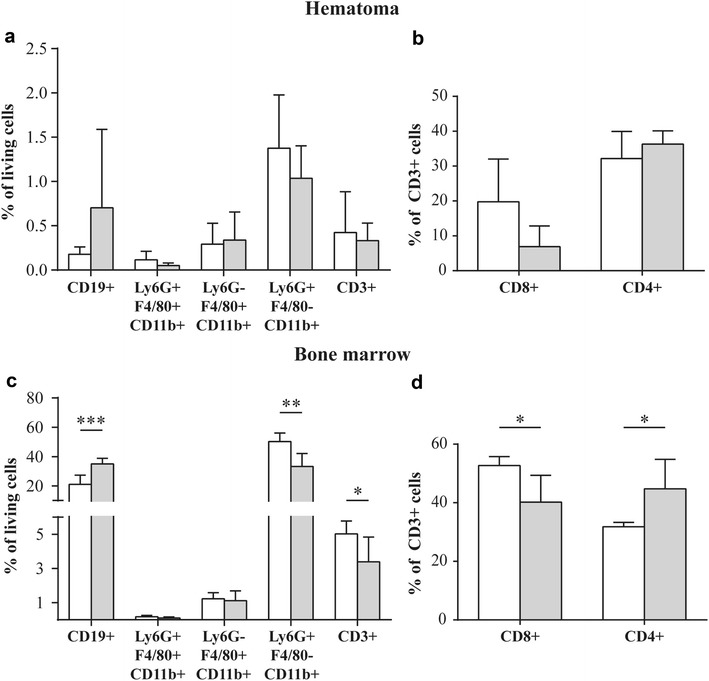
Immune-cell populations in fracture haematoma and bone marrow on day 1 after fracture analysed by flow cytometry. **a**–**d** Percents of living B-lymphocytes (CD19^+^), inflammatory monocytes (Ly6G^+^, F4/80^+^, CD11b^+^), macrophages (Ly6G^−^, F4/80^+^, CD11b^+^), neutrophils (Ly6G^+^, F4/80^−^, CD11b^+^), T-lymphocytes (CD3^+^), cytotoxic T-lymphocytes (CD3^+^, CD8^+^) and T-helper lymphocytes (CD3^+^, CD4^+^) in the fracture haematoma and bone marrow of sham-operated (*white bars*) and OVX-mice (*grey bars*). *p < 0.05, **p < 0.01, ***p < 0.001 for comparison between sham- and OVX-mice

**Fig. 3:**
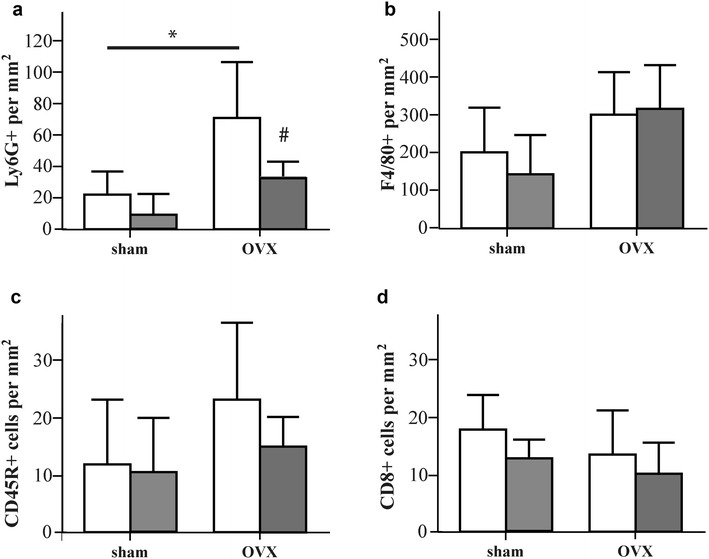
Number of immune cells in the fracture callus on day 3 after fracture analysed by immunohistochemistry. **a** Number of Ly6G-positive neutrophils per square mm of the periosteal callus. **b** Number of F4/80-positive macrophages per square mm of the marrow cavity. **c** Number of CD45R-positive B-lymphocytes per square mm of the periosteal callus. **d** Number of CD8-positive cytotoxic T-lymphocytes per square mm of the periosteal callus. *p < 0.05 for comparison between sham- and OVX-mice, ^#^ *p* < 0.05 for comparison between Mdk-Ab untreated (*white bars*) and treated mice (*grey bars*)

**Fig. 4:**
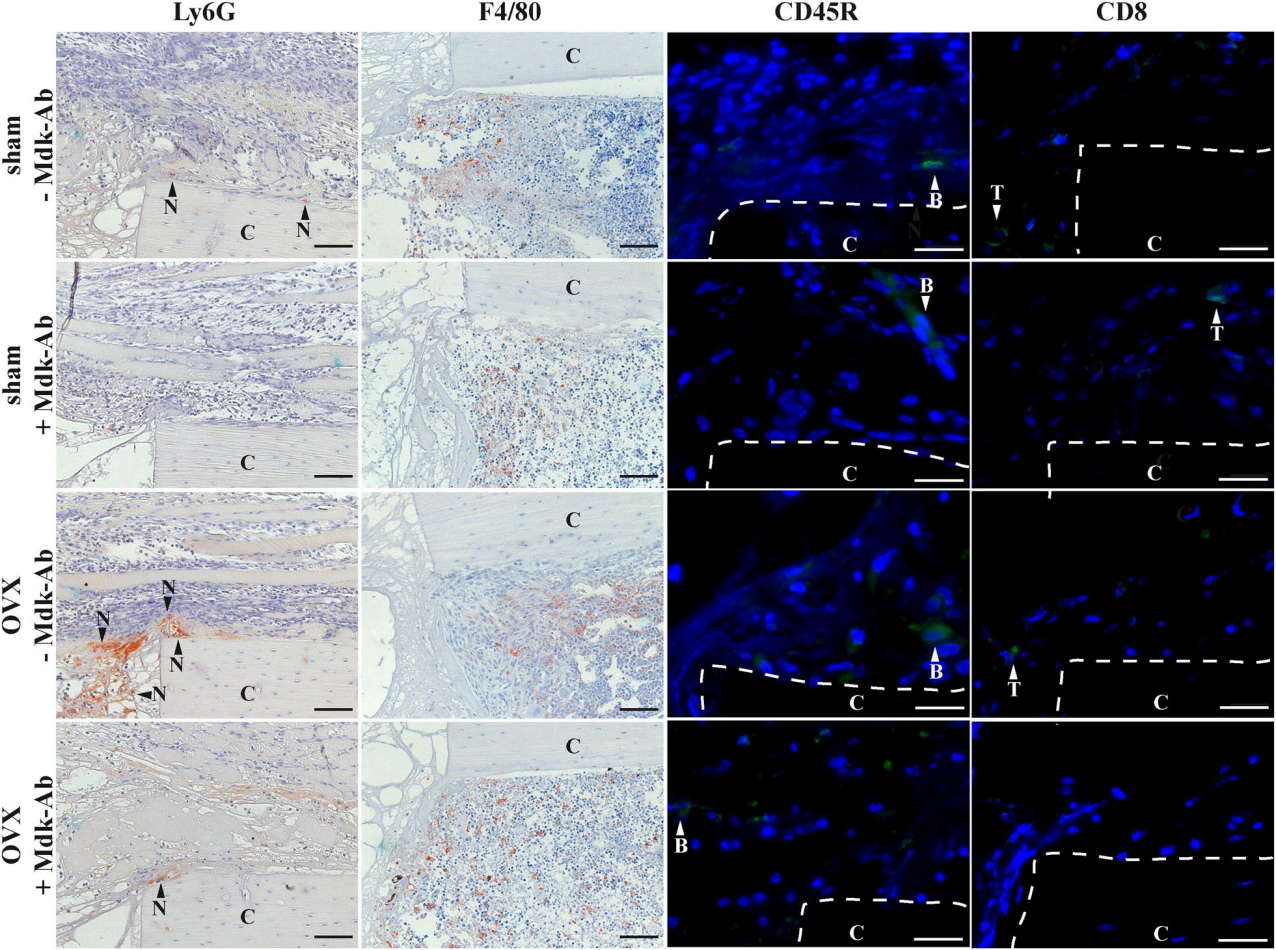
Immune-cell staining in the fracture callus on day 3 after fracture. Representative images of Ly6G^−^, F4/80^−^, CD45R^−^ and CD8-positive cells in the fractured femurs from sham-operated and OVX-mice treated with and without Mdk-Ab. The periosteal callus proximal to the osteotomy gap is shown. *N* neutrophil, *B* B-lymphocyte, *T* T-lymphocyte, *C* bone cortex. *Scale bar* = 100 µm for immunohistochemical staining and 25 µm for immunofluorescence staining

We analysed the presence of immune cells in the fracture haematoma on day one after surgery by FACS analysis (Fig. 2a, b). The cell populations of the innate and adaptive immune systems did not differ significantly between sham- and OVX-mice at this time point at the fracture site. However, OVX significantly increased the number of B-lymphocytes and reduced the number of T-lymphocytes in the bone marrow (Fig. 2c). Within the T-lymphocyte population, cytotoxic T-cells were significantly reduced, whereas T-helper cells were significantly increased (Fig. 2d). Furthermore, the numbers of inflammatory monocytes and macrophages were not affected, whereas the number of neutrophils was significantly decreased. Three days after fracture, immune cells in the fracture callus were evaluated by immunohistochemistry. OVX-mice displayed significantly greater numbers of neutrophils in the periosteal callus (Fig. 3a). Neutrophils were predominantly found in the fracture gap surrounding fibrous tissue as well as in the early periosteal fracture callus (Fig. 4). The number of macrophages, which were mainly located in the bone marrow near the osteotomy gap, did not differ significantly between the groups (Fig. 3b). There were no differences in the number of B-lymphocytes or cytotoxic T-lymphocytes, which were located in the periosteal fracture callus (Fig. 3c, d).

### OVX increased Mdk and IL-6 expressions in the fracture callus

**Fig. 5:**
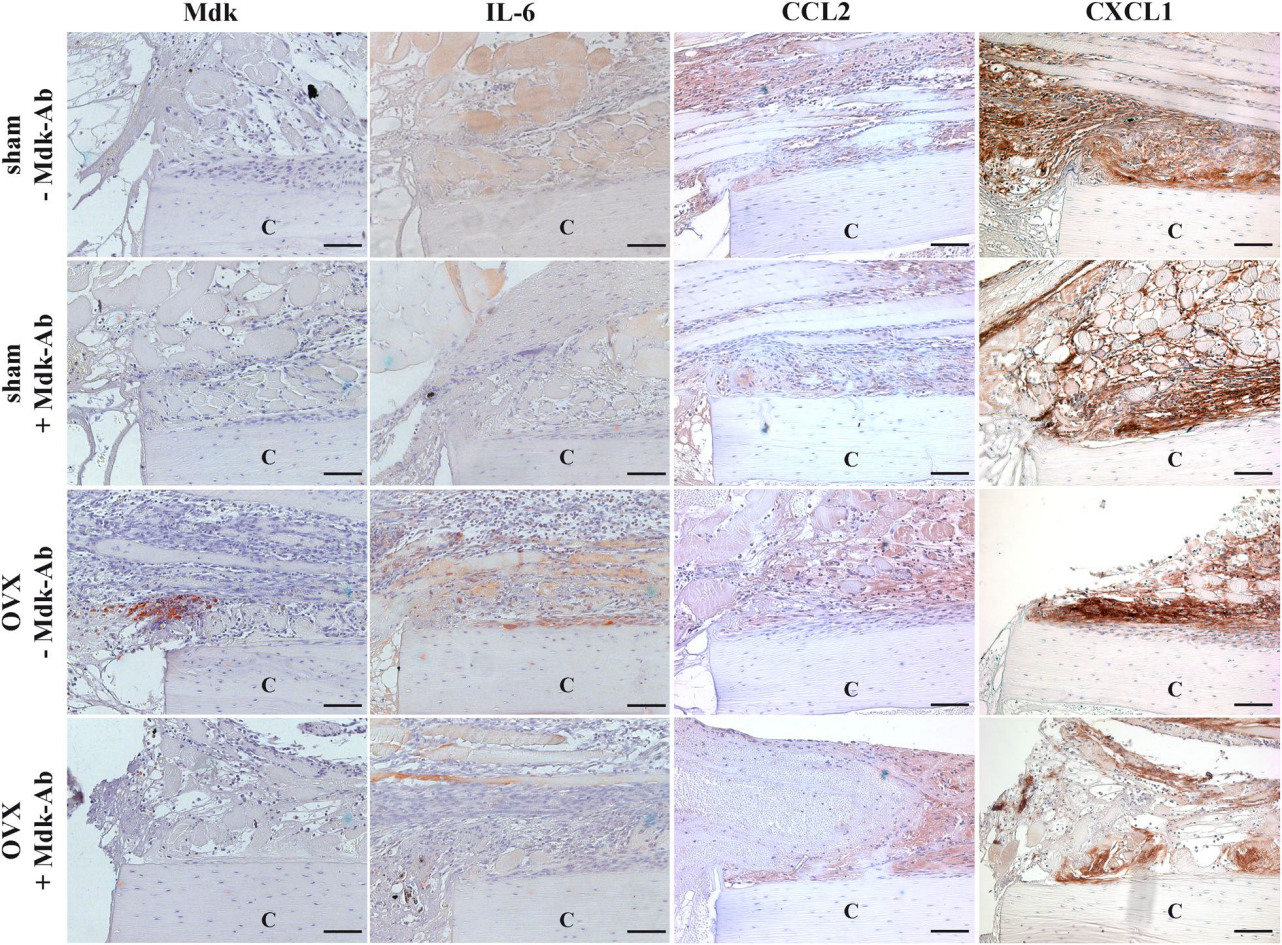
Cytokine expression in the fracture callus on day 3 after fracture. Representative images of Mdk-, IL-6-, CCL2- and CXCL1-immunostained sections of the fractured femurs from sham-operated and OVX-mice treated with and without Mdk-Ab. The periosteal callus proximal to the osteotomy gap is shown. *C* cortex. *Scale bar* = 100 µm

We investigated the role of Mdk during the inflammatory phase of fracture healing. Therefore, we first analysed the local expression of this protein in the callus by immunostaining. On day 3, Mdk was expressed in the fracture callus of OVX-mice (Fig. 5). The protein was located at the periosteal transition zone from haematoma tissue to soft fracture callus proximal to the osteotomy gap. Mdk was expressed less in sham-operated animals. Because it is known that Mdk expression is associated with IL-6 expression [40] and because IL-6 is a potent inducer for neutrophil recruitment [41], IL-6 expression was evaluated by immunostaining. In sham-mice, IL-6 was expressed in muscle tissue and endosteal cells, but was expressed less in periosteal cells, whereas OVX-mice showed increased expression in periosteal cells adjacent to the fracture gap (Fig. 5). We hypothesised that the up-regulation of Mdk expression after OVX may be involved in increased neutrophil numbers in the periosteal callus. Therefore, we treated mice with an inhibitory Mdk-Ab directly after surgery. Mdk-Ab-treated mice displayed significantly lower numbers of neutrophils in the fracture callus compared with non-treated mice (Fig. 3a), whereas the numbers of macrophages, B-lymphocytes and cytotoxic T-lymphocytes were not significantly altered (Fig. 3b–d). Furthermore, Mdk-Ab-treated OVX-mice exhibited lower IL-6 expression in the periosteal cells next to the fracture gap (Fig. 5). Protein expressions of the proinflammatory cytokines, CCL2 and CXCL1, were similar between the groups (Fig. 5).

## Discussion

It is well established that oestrogen affects the immune system and the severity of inflammatory disorders [42]. Because a balanced inflammatory response is considered to be crucial for proper bone healing [20], the question arises whether oestrogen-deficiency influences inflammation during early fracture healing. We analysed the immune cells in the bone marrow as the major source for migrating immune cells to the fracture haematoma [43]. Our results demonstrated that numbers of B-lymphocytes in the bone marrow were elevated in OVX-mice 1 day after fracture, whereas the number of bone marrow neutrophils and total T-lymphocytes were reduced but with an increased ratio of CD4^+^/CD8^+^ cells. It was previously reported that B-lymphocyte numbers increased in the bone marrow in response to OVX-induced oestrogen-deficiency [44–46]. Because B-lymphocytes are known to synthesise several inflammatory cytokines as well as recent finding suggesting that they are active regulators of the RANK/RANKL/OPG system, it was suggested that there is a strong association between increased numbers of B-lymphocytes and bone loss during menopause [47–49]. In general, the chronic inflammatory immune status in postmenopausal females is regarded to contribute to bone loss [50, 51]. It was also reported that oestrogen-deficiency considerably affects T-lymphocytes; however, data are conflicting, showing either an increase or decrease of T-lymphocytes in bone marrow and spleen [46, 52–54]. In agreement with our results, changes in the ratio of CD4^+^/CD8^+^ cells in the bone marrow were frequently observed in oestrogen-deficient animals [55–57]. Bone-marrow T-lymphocytes are suggested to contribute to the strong influence of the immune system on bone homeostasis and to modulate the bone-marrow environment in either an osteoclastogenic or an anti-osteoclastogenic manner, depending on the T cell subset [58]. Activated T-lymphocytes were shown to produce increased TNFα levels in response to oestrogen withdrawal, leading to increased bone resorption [52]. In particular, Th17 T-cells were demonstrated to link T-cell activation and osteoclast activation [59]. The roles of CD4^+^ and CD8^+^ T-cells during postmenopausal bone loss are strongly discussed. Depending on the manner in which these cells were activated, both subsets can either mediate osteoclastogenic or anti-osteoclastogenic effects [58]. In conclusion, the roles of T-lymphocytes in postmenopausal bone loss and chronic inflammation remain unclear. Even less is known about the contribution of bone-marrow neutrophils to osteoporotic bone loss. There are phenomenological studies showing increased [60], decreased [61] or unaltered [62] numbers of neutrophils after oestrogen withdrawal, but there are no mechanistic studies available.

Because it was shown that both B- and T-lymphocytes as well as neutrophils could affect fracture healing outcome [63–65], we evaluated the number of these cells in the early fracture haematoma. We did not find any differences between OVX- and sham-mice on day 1 after trauma, although the cell populations were different in the bone marrow. This finding indicates that the initial recruitment of inflammatory cells to the fracture callus was unaffected by oestrogen-deficiency. However, on day 3 after fracture, significantly more neutrophils were present in the periosteal callus of oestrogen-deficient mice, indicating a prolonged recruitment and/or an increased survival of neutrophils at the fracture site in the absence of oestrogen. In the literature, OVX is described to increase local activation of neutrophils after haemorrhagic shock [66], artery injury [67], lung damage [68] and during wound healing [69], although the mechanisms of interaction between oestrogen and neutrophils remain unclear. Our data suggest that the pro-inflammatory cytokine Mdk may be involved in the effects of oestrogen-deficiency on the inflammatory phase of fracture healing. It was shown previously that the promoter region of the *Mdk* gene contains oestrogen-responsive elements and that the expression of Mdk is enhanced in the kidney of diabetic, oestrogen-deficient mice [28]. In addition, oestrogen receptor α-deficient osteocytes displayed increased Mdk mRNA levels [27]. We demonstrated in a previous study, that Mdk serum levels were increased in oestrogen-deficient mice from day 3 to day 23 after fracture [23]. In the present study, we found increased local expression of Mdk in the fracture callus of OVX-mice on day 3 after fracture. Because Mdk is known to chemoattract both neutrophils and macrophages [33], we hypothesised that increased Mdk expression may be involved in the prolonged presence of neutrophils at the fracture callus in OVX-mice. Indeed, we found significantly decreased numbers of neutrophils after antagonising Mdk upon Mdk-Ab treatment. However, we did not detect significant changes in CXCL1 expression, one of the most important proteins for neutrophil recruitment. It is known from previous studies that Mdk-deficient mice displayed lower numbers of neutrophils and macrophages in the tubulointerstitium after ischaemic renal injury [32] and that Mdk-deficiency delayed the recruitment of macrophages to the fracture site during the regenerative phase of healing [70]. However, in the current study, we did not detect significant changes in the number of macrophages or the expression of monocyte chemoattractant protein 1 (CCL2). In addition, we did not detect changes in the numbers of B- or T-lymphocytes in the fracture callus, although it was demonstrated that Mdk regulated B-cell survival in vitro [71]. Therefore, the increased Mdk expression in the early fracture callus of oestrogen-deficient mice appears to predominantly affect the recruitment and survival of neutrophils.

In the literature, several pro-inflammatory cytokines are described to be involved in the increased severity of inflammatory disorders in oestrogen-deficient subjects. One of these cytokines is IL-6, which is known as a crucial factor for the recruitment of inflammatory cells [41, 72]. In addition, several studies demonstrated increased IL-6 expression after tissue injury in oestrogen-deficient mice [15, 16]. In the present study, we found higher IL-6 expression in the periosteal cells of OVX-mice. Indeed, it was shown previously that increased IL-6 expression was associated with greater numbers of neutrophils in the fracture callus after severe trauma [73]. Therefore, we suggest that increased IL-6 expression due to the lack of oestrogen might contribute to the increased number of neutrophils in the fracture callus of OVX-mice. In addition, there is some evidence in the literature that Mdk expression may be associated with IL-6 [40, 74].

In conclusion, our study demonstrated that oestrogen-deficiency significantly influenced the early inflammatory phase after fracture. Higher Mdk and IL-6 expression at the fracture site were associated with increased numbers of neutrophils in the callus.
